# The Use of Nanoparticles in Otoprotection

**DOI:** 10.3389/fneur.2022.912647

**Published:** 2022-06-29

**Authors:** Maurizio Barbara, Valerio Margani, Edoardo Covelli, Chiara Filippi, Luigi Volpini, Ola M. El-Borady, Maged El-Kemary, Saad Elzayat, Haitham H. Elfarargy

**Affiliations:** ^1^Department of Neuroscience, Mental Health and Sensory Organs, Faculty of Medicine and Psychology, Sapienza University, Rome, Italy; ^2^Otolaryngology Department, Liverpool University Hospitals NHS Foundation Trust, Liverpool, United Kingdom; ^3^Institute of Nanoscience and Nanotechnology, Kafrelsheikh University, Kafr El-Shaikh, Egypt; ^4^Otolaryngology Department, Faculty of Medicine, Kafrelsheikh University, Kafr El-Shaikh, Egypt

**Keywords:** nanoparticles, otoprotection, inner ear, cisplatin, dexamethasone

## Abstract

The inner ear can be insulted by various noxious stimuli, including drugs (cisplatin and aminoglycosides) and over-acoustic stimulation. These stimuli damage the hair cells giving rise to progressive hearing loss. Systemic drugs have attempted protection from ototoxicity. Most of these drugs poorly reach the inner ear with consequent ineffective action on hearing. The reason for these failures resides in the poor inner ear blood supply, the presence of the blood-labyrinthine barrier, and the low permeability of the round window membrane (RWM). This article presents a review of the use of nanoparticles (NPs) in otoprotection. NPs were recently used in many fields of medicine because of their ability to deliver drugs to the target organs or cells. The studies included in the review regarded the biocompatibility of the used NPs by *in vitro* and *in vivo* experiments. In most studies, NPs proved safe without a significant decrease in cell viability or signs of ototoxicity. Many nano-techniques were used to improve the drugs' kinetics and efficiency. These techniques included encapsulation, polymerization, surface functionalization, and enhanced drug release. In such a way, it improved drug transmission through the RWM with increased and prolonged intra-cochlear drug concentrations. In all studies, the fabricated drug-NPs effectively preserved the hair cells and the functioning hearing from exposure to different ototoxic stimuli, simulating the actual clinical circumstances. Most of these studies regarded cisplatin ototoxicity due to the wide use of this drug in clinical oncology. Dexamethasone (DEX) and antioxidants represent the most used drugs in most studies. These drugs effectively prevented apoptosis and reactive oxygen species (ROS) production caused by ototoxic stimuli. These various successful experiments confirmed the biocompatibility of different NPs and made it successfully to human clinical trials.

## Introduction

The inner ear is a sensitive organ that is liable to be insulted by many ototoxic stimuli, including drugs (cisplatin and aminoglycosides) and otoacoustic overstimulation. These stimuli damage the hair cells with progressive hearing loss, which may limit the administration of the required dose of life-saving anti-cancer drugs for an appropriate oncological medical protocol. Therefore, many studies were performed to prevent the ototoxic effect and minimize the associated hazards. Many drugs have been tried in the past, both systemically and locally, but they always had to face the natural inner ear barriers, showing ineffective hearing preservation. The success of nanotechnology in the construction of many successful drug delivery systems in several fields of medicine motivated the use of nanoparticles (NPs) in the field of otoprotection.

## The Limitations of Conventional Drug Delivery Systems Into the Inner Ear

Many barriers are limiting the effective systemic drug delivery into the cochlea. The blood–perilymph barrier is located in the modiolar vascular region (1, 2). The perilymph–endolymph barrier is most porous at the modiolar wall of the first and the second turns of both the scala vestibuli and the tympani. Moreover, the arterial supply to the labyrinth is sparse, thus requiring an increase in the dosage of intravenous or oral drugs to target the sensory inner ear cells (3). Thus, it is challenging to achieve therapeutic drug levels in the inner ear while minimizing toxicity and systemic side effects.

Drug delivery from the middle ear (after intratympanic injection) into the cochlea through the round window membrane (RWM) is another possibility, as this membrane is permeable to small molecules. In human, the thickness of the RWM is about 70 microns. The RWM consists of three layers: an outer epithelium, a fibrous connective tissue middle layer, and an inner epithelium surrounding the perilymphatic cavity. The middle collagenous layer may attenuate the transport. The permeability is mainly dependent on the drug particle size, charge, and solubility (4). The pharmacokinetics of RWM delivery may also be unpredictable due to variable clearance of drug through the Eustachian tube and variable patency of the cochlear aqueduct in human (5). Conversely, the RWM permeability can also be affected by inflammatory processes such as otitis media due to the scarring and fibrosis of granulation tissue hindering transport (6). However, prior studies have shown that positively charged molecules up to 500 nm in size can easily permeate the RWM (7). Positively charged substances (cationic) can pass more easily than negatively charged substances. They can bind to the negative cell surface glycoproteins, allowing easy passage through the RWM (8).

## The Emergence of NPs as a Drug Delivery System in the Field of Otology

There has been a growing interest in the otology community in exploring the potential use of NPs for intra-cochlear drug delivery. NPs' diameter ranges from 1 to 1,000 nm and can be used to stabilize and carry drugs to the cochlea. The use of nanocarriers emerged in the 1950's but has only been increasingly used for drug delivery within the last two decades (9). NPs can be engineered to allow non-invasive application, specific site targeting, and transporting across membranes such as RWM. Since NP-based drug delivery systems have already proven successful in many fields, such as oncology, it holds great potential for intra-cochlear drug delivery (10). However, NPs must be biocompatible within the cochlea, biodegradable, and non-toxic to the sensitive hearing cells to be used safely for treating hearing disorders. The need for safety poses a challenge in the NPs' research and development in otology. Recently, several types of NPs appear suitable for inner ear drug delivery, including poly lactic-co-glycolic acid (PLGA) NPs, magnetic NPs, lipid NPs, liposomes, polymers, hydroxyapatite NPs, and silica NPs (11).

## The Application of the NPs Drug Delivery System in the Otoprotection Field

One of the main auditory fields that need an evolution in its drug delivery system is otoprotection. Ototoxicity includes the cochlea (cochleotoxicity) and the vestibule (vestibulotoxicity) (12). Hearing loss and tinnitus are the main manifestations of cochleotoxicity with a substantial impact on communication. Hearing loss is bilateral, progressive, and irreversible, with impairment beginning at higher frequencies and progressing to lower ones (13). We found many previous studies that used NPs for otoprotection. In this review, we attempted to make an easy and simple analysis for otologists for a better understanding, which may allow for future human clinical applications.

## Previous Studies

According to the type of the ototoxic stimulus, we divided the NPs used for otoprotection into three main categories: against cisplatin, against gentamycin and kanamycin, and against acoustic trauma (Table 1).

**Table 1:** Summarization of the use of nanoparticles (NPs) in otoprotection.

	**References**	**Drug**	**Nanoparticle**	**Ototoxicity**	***In vitro***	***In vivo***	**Route of drug**	**Hearing evaluation**
1	Wang et al. (14)	Dexamethasone	A666-DEX-NP	Cisplatin	Yes	Guinea pigs	Bullostomy (Local)	ABR
2	Chen et al. (16)	Dexamethasone	DEX-loaded silk-polyethylene hydrogel	Cisplatin	Yes	Guinea pigs	Bullostomy (Local)	ABR
3	Martín-Saldaña et al. (25)	Dexamethasone α-tocopherol	Poly (VP-*co*-MTOS) Poly (VP-*co*- MVE)	Cisplatin	Yes	Wister rats	Bullostomy (Local)	ASSR
4	Petrova et al. (45)	Succinate	N-succinyl-chitin	Acoustic stimulation	No	Wister rats	Tail vein (systemic)	Otoacoustic emissions
5	Yüksel Aslier et al. (27)	Dexamethasone Genipin	Dexamethasone-loaded chitosan-based genipin-cross-linked hydrogel	Cisplatin	No	Guinea pigs	Intratympanic (local)	Otoacoustic emissions and ABR
6	Martín-Saldaña et al. (66)	Dexamethasone α-tocopherol Ibuprofen	Poly (VI-co-HEI) and Poly (VP-*co*-MVE) or Poly (VP-*co*-MTOS).	Cisplatin	Yes	Wister rats	Bullostomy (Local)	ASSR
7	Martín-Saldaña et al. (19)	6α-methylprednisolone α-tocopherol	Poly (VP-*co*-MTOS) Poly (VP-*co*- MVE)	Cisplatin	Yes	Wister rats	Bullostomy (Local)	ASSR
8	Panevin and Zhuravskii (49)	hydrocortisone	Poly-vinyl-pyrrolidone (povidone)	Acoustic stimulation	No	Wister rats	Intravenous (systemic)	Otoacoustic emissions
9	Gu et al. (30)	Astaxanthin (ATX)	Lipid-polymer nanoparticle	Cisplatin	Yes	Zebrafish Guinea pigs	Bullostomy (Local)	ABR
10	Cervantes et al. (32)	Dexamethasone Hydrocortisone	Stearic acid-based solid lipid nanoparticles	Cisplatin	Yes	NO	—	—
11	Ye et al. (34)	Dexamethasone Salvianolic acid B	Dexamethasone–Salvianolic Acid B Conjugates	Cisplatin	Yes	Zebrafish Guinea pigs	Transtympanic (Local)	ABR
12	Hou et al. (50)	Minocycline	SS-31 modified liposomes	Gentamycin	No	Zebrafish	—	—
13	Kayyali et al. (36)	Glutathione	Superparamagnetic iron oxide nanoparticles (SPIONs) entrapped within polymeric micelles	Cisplatin	Yes	No	—	—
14	Jung et al. (53)	Alpha-lipoic acid	Pluronic F-127 nanoparticles	Kanamycin	Yes	Mice	Bullostomy (Local)	ABR
15	Fernandez et al. (39)	Dexamethasone	Poloxamer hydrogel containing dexamethasone	Cisplatin	No	Guinea pigs	Intratympanic (local)	ABR
16	Sun et al. (40)	Dexamethasone	Polyethylene glycol-coated polylactic acid (PEG-PLA) stealth nanoparticles	Cisplatin	No	Guinea pigs	Bullostomy (Local)	ABR
17	Sun et al. (41)	Dexamethasone	Polyethylene glycol-coated polylactic acid (PEG-PLA)	Cisplatin	No	Guinea pigs	Intraperitoneal (systemic)	ABR
18	Salehi et al. (42)	Curcumin Dexamethasone	Nanoencapsulated Curcumin and Dexamethasone	Cisplatin	Yes	Guinea pigs	Intraperitoneal (systemic)	ABR

### Against Cisplatin

#### A666-Conjugated NPs Target Prestin of Outer Hair Cells

Wang et al. (14) created a novel dexamethasone (DEX) targeted NP composed of maleimide polyethylene glycol (PEG) and poly-lactic acid (PLA). To increase the effectiveness and delivery of DEX to the OHCs, they added A666 to the NP molecule. A666 is a peptide proved to have a high affinity to bind to the prestin, which has a high concentration in the OHCs. This resulted in a very specific active drug delivery by surface functionalization without affecting the molecular functioning (15). This was confirmed by the high internalization of A666-DEX-NP by the House Ear Institute-Organ of Corti 1 (HEI-OC1) compared to unconjugated DEX-NP. After cisplatin exposure, pretreated HEI-OC1 with A666-DEX-NP showed better viability than pretreatment with DEX only or unconjugated DEX-NP. For RWM application of A666-DEX-NP in guinea pigs *via* bullostomy,a postauricular incision was made, and the muscle was dissociated *via* blunt dissection and retracted until exposure to the auditory bulla. A hole of 2–3 mm diameter was drilled on the bulla to visualize the round window niche. The drug was applied to the RWM using a microsyringe. The guinea pigs were fixed in this position for 30 min. The bulla opening was sealed with dental cement, and the incision was closed with sutures. The local drug administration 3 days before intraperitoneal administration of cisplatin was effective in the partial preservation of hearing, especially at 4, 8, and 16 kHz frequencies using auditory brainstem response (ABR). Also, it was associated with higher preservation of the OHCs.

#### Dexamethasone-Loaded Injectable Silk-PEG Hydrogel

Chen et al. (16) used silk-PEG hydrogel as a vehicle for DEX delivery into the cochlea through RWM administration. Silk hydrogel is a natural protein tried for local, sustained drug release due to its versatility, biocompatibility, and biodegradation (17). The drug-loaded silk hydrogel can be attached to the RWM directly, allowing penetration of the stratum corneum′s outermost layers into the inner ear and staying in the perilymph for 14 days (18). The silk-PEG hydrogel did not cause toxicity to HEI-OC1 cells at a concentration below 60 ng/ml. Pretreatment with DEX-silk hydrogels exhibited enhanced cytoprotective effects by long-term sustained release of DEX in the HEI-OC1 cells exposed to cisplatin by attenuating the reactive oxygen species (ROS) levels. In a guinea pigs' module, the mean ABR threshold test indicated that the DEX-silk hydrogel partially protected the auditory functions compared to only DEX on the fifth day after cisplatin injection.

#### 6α-Methylprednisolone-Loaded NPs

Martín-Saldaña et al. (19) prepared surfactant-free polymeric micellear nanoaggregates that carried 6 α-methylprednisolone (20) and used these nanoaggregates for otoprotection. The multi-micellar nanoaggregates composed of a hydrophilic segment based on N-vinyl pyrrolidone, which can avoid the reticuloendothelial system, and a hydrophobic segment formed of a methacrylic derivative of vitamin E (MVE) or a methacrylic derivative of α-tocopheryl succinate (MTOS). The hydrophobic core allowed the encapsulation of poorly water-soluble molecules such as the 6α-methylprednisolone. MVE formulations were more cytotoxic than MTOS formulations. The pretreatment of the HEI-OC1 by the prepared NPs with concentrations <2 mg/ml was effective in maintaining the cellular viability after cisplatin exposure. Moreover, the prepared NPs were effective for the partial protection of hearing in the Wister rats if they were exposed to cisplatin with an upper hand for MVE NPs. Also, NPs were preferentially accumulated in the inner hair cells (IHCs) than in OHCs of the basal turn of the cochlea 2 h after middle ear administration.

#### pH-Sensitive Polymeric NPs

Martín-Saldaña et al. (19) combined two pseudo-block polymers to form a micelle with improved bioavailability and drug loading capacity to encapsulate three different hydrophobic drugs. These drugs included α-tocopheryl succinate (α-TOS), ibuprofen, and DEX. The cytoprotective effects of these drugs are related to their ability to prevent apoptosis caused by cisplatin and inhibit ROS production (21–23). Furthermore, they depended on the pH-responsive drug delivery nano-system to increase the concentration of the used drugs in the targeted cells. The polymeric NPs are frequently uptook by endocytosis within endosomal and lysosomal compartments whose pH is <5.5 (24). Pretreatment with the prepared NPs protected the HEI-OC1 against cisplatin with a downregulation of caspase 3/7 expression, lower interleukin (IL)-1β release, and lower intracellular ROS accumulation. Moreover, steady-state responses [acoustic steady-state response (ASSR)] test measurements showed the ability of the prepared NPS to protect Wister rats against cisplatin ototoxicity.

#### Polymeric NPs Loaded With DEX or α-TOS

Martín-Saldaña et al. (25) used copolymer NPs to carry DEX or α-tocopherol (α-TOS) in their hydrophobic core as these drugs are poorly soluble in water. They used these NPs for cytoprotection against cisplatin. The succinate form of α-TOS can prevent apoptosis caused by cisplatin due to its ability to monopolize the mitochondria respiratory chain, regulate ROS production, decrease caspase 3/7 activity, and release IL-1 (26). Cytotoxicity of DEX-loaded and α-TOS-loaded NPs was tested using HEI-OC1, and the results demonstrated that only the highest concentration (2.0 mg/ml) of DEX-NP was cytotoxic after 24 h (viability < 70%). α-TOS-loaded formulations were more cytotoxic than DEX formulations. Pretreatment with DEX-NP or α-TOS-NP was effective in protecting HEI-OC1 if they were exposed to cisplatin. On the same side, steady-state auditory responses (ASSR) test results revealed the ability of DEX-NP and α-TOS-NP to protect the auditory functions of the Wister rats against cisplatin.

#### Dexamethasone-Loaded Chitosan-Based Genipin Cross-Linked Hydrogel

Yüksel Aslier et al. (27) prepared DEX-loaded CBGCH NPs in the form of thermogel and used it for otoprotection. Genipin is a natural cross-linking reagent with cytoprotective, potential anti-inflammatory, and anti-angiogenic effects. Also, genipin has inhibitory effects on lipid peroxidation and nitric oxide synthesis (28). The positively charged nature of chitosan provides interesting properties with respect to interaction with oppositely charged biological materials such as cells, membranes, negatively charged mucosal surfaces, and other macromolecules such as DNA. Thus, according to the Donnan effect, Chitosan has easier diffusion/penetration into the cochlea (29). Intratympanic injection of CBGCH in guinea pigs effectively protected the cochlea against the cytotoxic effect of intraperitoneal administration of cisplatin. This was confirmed by distortion product otoacoustic emissions (DPOAEs) and ABR measurements. Moreover, the scanning electron microscope (SEM) analysis showed that stereocilia of IHCs and OHCs were preserved because of the cytoprotective effect of CBGCH.

#### Astaxanthin-Loaded Polymer-Lipid Hybrid NPs

Gu et al. (30) prepared lipid-polymer NPs (LPN) and used them to carry and protect astaxanthin (ATX). The administration of ATX can prevent the pathogenesis of ROS-related conditions effectively. However, the high lipophilicity and thermolability of ATX limit its antioxidant efficacy in human (31). Using ATX-LPN overcomes this limitation by allowing homogenous dispersions in an aqueous solution and strong interactions with hair cells *via* electrostatic effects. The pretreatment of the HEI-OC1 by ATX-LPN has a higher cytoprotective ability against cisplatin than ATX molecules. ATX-LPN can suppress the cisplatin-induced release of pro-apoptotic proteins, cleaved-caspase 3/9, and cytochrome-c. Also, it can increase the expression level of the anti-apoptotic proteins Bcl-2. On the same side, ATX-LPN showed great efficacy in the otoprotection of Zebrafish hair cells against cisplatin. This efficacy was also obvious in guinea pigs if they were exposed to cisplatin 3 days after RWM application of ATX-LPN, especially in the higher and the ultrahigh frequencies. ATX-LPN can suppress the expression caspase-3 rescuing the OHCs.

#### Solid Lipid NPs Loaded With Glucocorticoids

Cervantes et al. (32) incorporated glucocorticoids (DEX and hydrocortisone) into stearic acid-based solid lipid NPs (SLNs) and used them for otoprotection. Lipid NPs are biodegradable and can deliver hydrophilic and lipophilic drugs. SLNs, a new generation of submicron-sized lipid particles, can be an alternative to the existing lipid nanocarriers. Additional advantages are the well-established, low cost, easy production method and the microemulsion, in which organic solvents are avoided (33). The prepared NPs were efficiently incorporated and biocompatible with the HEI-OC1 cells. These NPs were more effective in the otoprotection of the HEI-OC1 cells against cisplatin than the free DEX and hydrocortisone molecules.

#### Dexamethasone–Salvianolic Acid B (SAL) Conjugates

Ye et al. (34) conjugated DEX (hydrophobic) to a hydrophilic drug (SAL) *via* an ester bond to form a nanomolecule that was used for otoprotection. SAL has been widely used to treat cardiovascular and cerebrovascular diseases. The protective effects of SAL appear to be mediated *via* anti-apoptotic, antioxidative, and anti-inflammatory functions (35). The pretreatment with the prepared NPs effectively maintained the viability of the HEI-OC1 cells if exposed to cisplatin. This cytoprotective capability of the DEX-SAL NPs was also shown in the Zebrafish hair cells against cisplatin. Moreover, pretreatment of guinea pigs with DEX-SAL NPs effectively protected the ipsilateral and contralateral cochleae against the ototoxic effect of cisplatin without obvious inflammatory responses. The cochlear aqueduct may be the potential transfer route to the contralateral ear.

#### Superparamagnetic Iron Oxide NPs Entrapped Within Polymeric Micelles

Kayyali et al. (36) prepared SPIONs encapsulated within the hydrophobic core of micelles formed of di-block copolymer conjugated to a glutathione diethyl ester to enable the sequestration of cisplatin. Glutathione is a thiol-containing tripeptide and an extremely important cellular antioxidant. Glutathione plays a major role in cellular cisplatin resistance by forming highly stable complexes with cisplatin (37). The micelles are designed to be extracted from the inner ear once they have sequestered the cisplatin defused into the inner ear. This should prevent the development of any potential long-term adverse interactions between the micelles and the cochlear tissue. In their experiments, this was done by an electromagnet which was applied to the samples for one hour to remove the majority of the micelles. The remaining micelles were then removed using a 50-k centrifuge filter (38). The cochlear organotypic culture study showed that the thiol micelles did not alter the cochlear hair cell morphology nor cause any visible damage, even after 72 h incubation. Thus, the micelles are safe to use *in vivo* inner ear studies. The pretreatment of HEI-OC1 cells with the prepared NPs maintained cellular viability after cisplatin exposure.

#### Sustained-Exposure DEX Formulation OTO-104

Fernandez et al. (39) used poloxamer hydrogel containing DEX (OTO-104) in the otoprotection by the sustained release of DEX. OTO-104 consisted of a sterile suspension containing DEX in 16% poloxamer 407. Poloxamers are synthetic triblock copolymers of poly (ethylene oxide)-b-poly (propylene oxide)-b-poly (ethylene oxide) (PEO-PPO-PEO) forming micelles of a nanosize used as drugs nanocarriers. In guinea pigs, a single intratympanic administration of 6% OTO-104, but not of lower doses, almost completely protected against acute cisplatin ototoxicity compared to free DEX. OTO-104 was also very effective in protecting the cochlea despite the chronic administration of cisplatin (3 injections of cisplatin at a weekly interval).

#### Dexamethasone Encapsulated in PEG-PLA (Local)

Sun et al. (40) encapsulated DEX in PEG-coated polylactic acid NPs and used them for otoprotection. Coumarin 6-labeled NPs placed onto the RWM of guinea pigs penetrated the RWM effectively and accumulated in the organ of Corti, the stria vascularis, and the spiral ganglion cells after 1 h of application. The DEX-NPs locally applied onto the RWM of guinea pigs by a single-dose administration continuously released DEX for 48 h, which was significantly longer than the free DEX that was cleared out within 12 h after administration at the same dose. Further functional studies showed that locally administrated single-dose DEX-NPs effectively preserved OHCs in guinea pigs after cisplatin administration and thus significantly attenuated hearing loss at 4 and 8 kHz frequencies compared to the effect of free DEX formulation. The histological evaluation indicated that the administration of DEX-NPs did not induce local inflammatory responses.

#### Systemic Administration of DEX-Loaded NPs

Sun et al. (41) evaluated the potential protective effect of the systemic DEX encapsulated in PEG-PLA NPs (DEX-NPs) against cisplatin-induced hearing loss. Free DEX or DEX-NPs were administered intraperitoneally to guinea pigs 1 h before cisplatin injection. ABR threshold shifts were evaluated 1 day before and 3 days after cisplatin administration. Cochlear morphology was assessed to evaluate inner ear injury induced by cisplatin exposure. A single dose of DEX-NPs 1 h before cisplatin treatment resulted in effective preservation of the cochlear functional and structural properties, equivalent to the effect of multi-doses (3 days) of free DEX injections.

#### Nanoencapsulated Curcumin and DEX

Salehi et al. (42) nanoencapsulated DEX and curcumin and used the formed NPs for otoprotection. Curcumin has antioxidant, anti-inflammatory, and antineoplastic activities. Regardless of the administration route, the bioavailability of curcumin is low due to hydrolytic degradation, chemical instability in alkaline pH, and rapid intestinal and hepatic metabolism/elimination. Recent nanoencapsulation of curcumin has provided a strategy for increasing its solubility and stability in aqueous media to reach many cellular compartments, including inner ear cells, which are the main target of cisplatin ototoxicity (43, 44). Pretreatment with the prepared NPs effectively preserved the viability of HEI-OC1 cells after cisplatin administration. In guinea pigs, the pretreatment with encapsulated DEX and curcumin prevented the ototoxic effect of cisplatin and preserved hearing in all frequencies. This effect was associated with lowering superoxide dismutase (SOD) and catalase activities.

### Against Acoustic Trauma

#### N-Succinyl-Chitin NPs

Petrova et al. (45) prepared N-succinyl-chitin NPs and tried them for otoprotection. Exogenous administration of succinate under anaerobic conditions lowers the concentrations of lactate, pyruvate, and citrate while maintaining necessary ATP production with cytoprotective and antihypoxic effects (46). The main disadvantage of succinate-containing drugs is their low concentrations in target cells. The exogenous succinate ions undergo an untargeted competitive utilization by organs not involved in the pathological process. Also, histo-hematic barriers limit the application of these low molecular weight drugs (47). A chitin dispersion can increase the cochlear biodistribution of succinate due to the large surface area of their fibers (48). N-succinyl-chitin NPs effectively protected the cochlea against an acute acoustic trauma in Wister rats. These NPs showed a better penetration into the cochlea, earlier restoration of the cochlear function at the studied frequencies, a more prolonged presence in the blood circulation, and a more prolonged cytoprotective action.

#### Hydrocortisone Immobilized on Povidone NPs

Panevin Zhuravskii (49) used hydrocortisone suspension, in which particles from synthetic organic polymer, polyvinylpyrrolidone (povidone), served as the dispersed transporting system. DPOAE measurements revealed the prepared NPs' ability to protect the cochlea against the acute pathology induced by a single acoustic stimulation of 5 kHz frequency and 110–112 dB intensity in Wister rats. The maximum otoprotection effect was more obvious 24 h after exposure to the acoustic stimulus.

### Against Gentamycin and Kanamycin

#### SS-31-Modified Liposomes for Improved Protective Efficacy of Minocycline

Hou et al. (50) modified the surface of liposomes by adding a mitochondria-targeting tetrapeptide (SS-31). SS-31 modified NPs (SS31-Lp) have a protective efficacy by decreasing the activity of mitochondrial channels, which play a critical role in converting mechanical stimuli into an electrical response to underlie the sensations of sound (51). This protective effect was enhanced by adding MC, a tetracycline derivative, which inhibits the mitochondrial Ca^2+^/Fe^2+^ uniporter and improves energy coupling in mitochondria at low concentrations. MC has neuroprotective effects against neurodegenerative diseases, such as amyotrophic lateral sclerosis and Huntington's disease. MC attenuated noise-induced hair cell loss in a Guinea pig model (52). SS31-Lp/MC NPs were biocompatible at low concentrations. Pretreatment with SS31-Lp/MC significantly increased the number of surviving Zebrafish hearing cells upon chronic exposure to gentamycin. In contrast, these NPs were ineffective in the otoprotection in the acute exposure to gentamycin.

#### Alpha-Lipoic Acid Loaded Pluronic F-127 NPs

Jung et al. (53) prepared NPs by encapsulating ALA into Pluronic F-127, which acts as a hydrophilic shell component of the formed NPs. ALA is an antioxidant that prevents ototoxicity by activating the Nrf2/HO1 antioxidant pathway (54). Pluronic F-127 contains two PEG groups and is approved by the United States Food and Drug Administration for intravenous infusion (55). ALA-NPs were biocompatible when used on HEI-OC1 cells. Also, pretreatment with ALA-NPs effectively protected HEI-OC1 cells against kanamycin ototoxic effect compared to free ALA molecules. This cytoprotective effect was associated with nuclear translocation of Nrf2 and increased levels of antioxidant proteins, including HO1, SOD1, and SOD2. When ALA-NPs were injected into the mouse middle ear cavity, the hearing ability was significantly preserved after the induction of ototoxicity of kanamycin, compared to the free ALA molecules.

## Discussion

Transtympanic injection of NPs is conceptually the best method for delivering drugs for inner ear disorders. Subsequently, the drug would be transferred *via* the oval window or round window membranes. NPs can stabilize drug molecules and achieve site-specific targeting with a controlled release of the drug molecules to the site of action. The targeting system's capability of NPs allows their widespread application in drug delivery systems, including hydrogels and scaffolds, allowing for a higher concentration of drugs to be delivered. After its administration, NPs can distribute in the perilymph and endolymph compartments and can be targeted to a specific site of interest. Development of targeting capacity can be done by altering surface chemistry with functional groups and ligands (56, 57).

## Causes of Ototoxicity

According to the studies included in the present review, the main clinical issues involved in cochleotoxicity are due to cisplatin (the most common, 14 studies), acoustic trauma (two studies), and aminoglycosides, including gentamycin and kanamycin (two studies).

### Cisplatin

Cisplatin is a highly efficient chemotherapeutic agent used against solid tumors, including the head and neck, lung, ovary, bladder, and testicles. One of the main side effects of cisplatin is ototoxicity which develops as a bilateral, gradual, and severe hearing loss, with a prevalence of 40–80% in adult patients with cancer and >50% in pediatric patients with cancer after cisplatin administration. This impairment can lead to a multifaceted decrease in the quality of life and notably impact pediatric patients' social and scholarly development (58). Cisplatin induces apoptosis of the inner ear cells by binding to DNA, ROS generation, increased lipid peroxidation, Ca^2+^ influx, and other inflammation events. Hearing impairment begins at the high frequencies and progresses to mid-range when the patient receives doses higher than 100 mg/m^2^ of the body surface. Patients who receive ultrahigh doses of cisplatin (150–225 mg/m^2^) show hearing loss in the high and ultra-high frequencies in 100% of cases. Rapid intravenous bolus injections, high cumulative doses, pre-existing hearing loss, renal insufficiency, anemia, hypoalbuminemia, and prior cranial irradiation can enhance cisplatin toxicity (59, 60). González-García et al. found a significant increase in total SOD activity and the expression of caspase-3/7 and caspase-9 in whole cochlear extracts related to an antioxidant response on the seventh day after a single dose of 5 mg/kg cisplatin administration. Depletion in endogenous antioxidant enzymes like SOD, catalase, glutathione peroxidase, and glutathione reductase were observed in animals that have received ototoxic doses of cisplatin (16 mg/kg), leading to ROS accumulation and resulting in apoptosis (61).

### Acoustic Trauma

An acoustic trauma initiates vascular stria injuries, especially to the types II and IV fibrocytes pericytes with subsequent rupture of their connections to endothelial cells. This causes endotheliocyte degeneration and endothelium desquamation, leading to vasoconstriction, hyperemia, and edema with subsequent apoptosis of the hair cells (62).

### Aminoglycosides

Aminoglycosides are important antibiotics in the current clinical practice because of their potency in treating life-threatening infections. Thus, they are widely used, despite the risk of ototoxicity that develops in up to one in four treated patients. Administration of clinically relevant doses of aminoglycosides damages the OHCs, which are known to execute the mechanical amplification of sound entering the ear. Polycationic aminoglycosides rapidly and specifically enter hair cells *via* mechano-transduction (MET) channels, where they localize to a variety of subcellular organelles, including mitochondria. The mitochondria rapidly swell in response to aminoglycoside exposure, eventually leading to mitochondrial dysfunction and cell apoptosis through overproduction of ROS and other apoptosis-inducing factors (63).

## Mechanism of Action of the Used Drugs

One of the main drugs′ classes used in the otoprotection in the current review (13 studies) is represented by corticosteroids, mostly DEX. DEX, a synthetic steroid analog, is widely given to treat inner ear diseases, including sudden idiopathic sensory neural hearing loss and Meniere's disease. Recently, DEX was confirmed as a candidate for otoprotection based on its ability to upregulate the antioxidant enzyme activity and activate the cell survival pathways. DEX binds to the intra-cochlear glucocorticoid receptor. It then inhibits the signal transduction pathway that mediates inflammation and induces apoptosis by modulating nuclear factor kappa B (NF-κB) and inhibiting ROS production in the inner ear. However, systemic glucocorticoids are limited by the blood–cochlear barrier that restricts drugs from leaving the circulation and gaining access to the inner ear cells, resulting in the lack of functional otoprotective activity. Furthermore, the systemically-administered glucocorticoids can diminish the tumoricidal activity of cisplatin *via* downregulating apoptotic genes in tumor cells. These problems have been mostly solved using the NP-based drug delivery systems (64, 65).

## Analysis of the Included Studies

In “*in vitro*” experiments, the HEI-OC1 system was used to determine the cells' viability and the molecular changes after applying the NPs with or without the applied ototoxic stimuli. At the same time, animal models were commonly used for “*in vivo*” experiments. Most of the included studies cared about the biodegradation (the ability of the body to eliminate the drug to prevent its accumulation and the consequent toxicity) of NPs used for the otoprotection, especially those with detailed preparation and characterization. The prepared NPs completely disappeared from the cochlea and the perilymph in a time longer than the conventional drugs (without NPs), allowing longer action and more effect. The efficiency of the used drug-NPs molecules was assessed functionally by auditory evaluation (ABR, otoacoustic emissions, or ASSR) with or without histological evaluation. In most studies, the used NP drug molecules effectively preserved hearing in most frequencies but with less effect at the high frequencies since the ototoxic stimuli (especially cisplatin) mainly affected the OHCs of the basal part cochlea. Characterization was done in most of the included studies, which allows for a better understanding of the nature of the use of NPs. This characterization confirmed that the ideal NPs for local drug delivery to the inner should be <500 nm in diameter, with negative zeta potential, no aggregation, a spherical shape, good encapsulation, storage stablity, sustained release for a long time, not cytotoxicity for the hair cells, and no unwanted change of the associated drug. Negative zeta potential enhances the molecule's stability without aggregation, maintaining the small-sized particles with a better action of the hair cells. It enhances adhesion and absorption with less cytotoxic effects than the positive zeta-potential molecules. Although most of the used NPs were given locally, some others were also given systemically. The studied systemic NPs used for cisplatin did not show a decrease in the effectiveness of cisplatin against the tumor cells. However, we believe more studies are needed to prove this. The combination of more than one drug in some studies is a promising way to increase the effectiveness of the used NPs in future experiments with the hope of a better outcome. NPs used for otoprotection can be classified into two main categories. The first category included inorganic NPs as SPIONs. In comparison, the other category included organic NPs such as polymers (surfactant-free polymeric micellear nanoaggregates carrying 6 α-methylprednisolone), PEG (encapsulated DEX in PEG-coated polylactic acid NPs), and liposomes (SS-31 modified NPs). According to our knowledge, there are no current human trials to use NPs in otoprotection. In this review, we aimed to highlight this promising direction in otoprotection, which may open the gate for human trials.

## Disadvantages of NPs

Although the effective capability of the NPs for the otoprotection has been proven, there are some associated disadvantages. The cost of their preparation is high, and they needs a longer time than the conventional drugs. Their preparation needs a multidisciplinary team with special skills and experiences in nanotechnology. More *in vivo* and *in vitro* experiments are needed to ensure their safety. Although the safety of their use is shown in different studies, they are still associated with some toxicity above a certain concentration. Human use may be accompanied by other side effects or a decrease in the effectiveness of cisplatin against the tumor cells.

## Conclusions and Future Application

The NP-drug delivery system in otoprotection seems to be very effective. The included studies considered the biocompatibility of the used NPs by *in vitro* and *in vivo* experiments. In most studies, the NPs were safe without significantly decreasing cell viability or animal ototoxicity. Many nano-techniques were used to improve the drugs' kinetics and efficiency. These techniques included encapsulation, polymerization, surface functionalization, and enhanced drug release. This resulted in an improvement in drug transmission through the RWM and an increase in the intra-cochlear drug concentration with prolonged persistence. In all studies, the fabricated drug-NPs effectively preserved the inner ear hair cells and the functioning hearing from exposure to different ototoxic stimuli, simulating the actual clinical circumstances. Most of the included studies gave a detailed guide for the preparation, characterization, and mechanism of the used NPs at the cell level or even at the ultra-cellular level. Most of these studies used cisplatin as the ototoxic stimulus due to its wide use in oncology. DEX and antioxidants, mostly used in the mentioned studies, proved effective in preventing apoptosis and ROS production caused by ototoxic stimuli. These various positive experiments confirm the biocompatibility of different NPs and open the path for human clinical trials. Moreover, NPs proved successful for otoprotection compared to conventional drug delivery methods. A combination of two or more of the mentioned techniques may represent the best way to find the perfect NPs in otoprotection.

## Author Contributions

All authors listed have made a substantial, direct, and intellectual contribution to the work and approved it for publication.

## Conflict of Interest

The authors declare that the research was conducted in the absence of any commercial or financial relationships that could be construed as a potential conflict of interest.

## Publisher's Note

All claims expressed in this article are solely those of the authors and do not necessarily represent those of their affiliated organizations, or those of the publisher, the editors and the reviewers. Any product that may be evaluated in this article, or claim that may be made by its manufacturer, is not guaranteed or endorsed by the publisher.

## Abbreviations

NPs: nanoparticles
RWM: round window membrane
PLGA: poly lactic-co-glycolic acid
DEX: dexamethasone
PEG: polyethylene glycol
PLA: poly-lactic acid
OHCs: outer hair cells
HEI-OC1: House Ear Institute-Organ of Corti 1
ABR: auditory brainstem response
MVE: methacrylic derivative of vitamin E
MTOS: methacrylic derivative of α-tocopheryl succinate
IHCs: inner hair cells
α-TOS: α-tocopheryl succinate
ROS: reactive oxygen species
IL: interleukin
ASSR: acoustic steady-state response
CBGCH: chitosan-based genipin-cross-linked hydrogel
DPOAEs: distortion product otoacoustic emissions
SEM: scanning electron microscope
LPN: lipid-polymer nanoparticles
ATX: astaxanthin
SLNs: solid lipid nanoparticles
SAL: salvianolic acid B
SPIONs: superparamagnetic iron oxide nanoparticles
SS31: mitochondria-targeting tetrapeptide
MC: minocycline
ALA: alpha-lipoic acid
Nrf2: nuclear factor erythroid 2-related factor 2
SOD: superoxide dismutase
MET: mechano-transduction
NF-kB: nuclear factor kappa B.
